# Clinical outcomes of transcatheter closure of congenital coronary artery fistula in 28 cases

**DOI:** 10.34172/jcvtr.31823

**Published:** 2024-06-25

**Authors:** Azin Alizadehasl, Ata Firouzi, Zahra Khajali, Ehsan Khalilipur, Zahra Hosseini, Hanieh Nezhadbahram, Tayebe Mohamad Gholizad, Fateme Amini, Tahere Sahraee, Shoeib Dehbandi, Seyed Ehsan Parhizgar

**Affiliations:** ^1^Cardio-Oncology Research Center, Rajaie Cardiovascular Medical and Research Center, Iran University of Medical Sciences, Tehran, Iran; ^2^Cardiovascular Intervention Research Center, Rajaie Cardiovascular Medical and Research Center, Iran University of Medical Sciences, Tehran, Iran; ^3^Rajaie Cardiovascular Medical and Research Center, Iran University of Medical Sciences, Tehran, Iran

**Keywords:** Coronary artery fistula (CAF), Congenital heart disease, Percutaneous coronary intervention

## Abstract

Most cases of congenital coronary artery fistula (CAF) resolve spontaneously, symptomatic patients with severe shunting require surgical intervention. Our aim is to evaluate success rate and outcome of CAFs treatment using transcatheter interventional methods.This retrospective study conducted on 28 CAF patients who were referred to Rajaie Cardiovascular Medical and Research Center in Tehran between 2015 and 2020. Baseline characteristics were collected by assessing hospital records, and patients were followed up annually for long-term evaluation. All of 28 patients gone throughtranscatheter closure of CAF. In 23 patient’s it was proximal type (82.1%) and in 5 patients was distal type (17.9%). In 11 patients, the fistula originated from the RCA (39.3%) and in 11 patients, it originated from the LAD and Diagonal. Most common drainage site was the pulmonary artery (82.1%). Coil used in 23 patients(82.1%). PDA occluder (7.1%) for 2 patients. VSD occluder for one patient (3.6%) and VSD+PDA occluder combination was used for one patient (3.6%). Procedure failure was in only one patient. Non-significant remaining shunt in the injection immediately after the procedure was seen in 4 patients (14.3%), which was reduced during the follow-up. None of the patients had significant shunt or clinical symptoms during long-term follow-up. As for complications, fistula dissection occurred in only one patient.The transcatheter interventional approach for the treatment of CAFs leads to favorable long-term results.

## Introduction

 Congenital coronary artery fistula (CAF) is an abnormal connection of a coronary artery to the cardiac chambers or vascular structure located near the heart. CAF is a rare congenital anomaly with a reported incidence of 0.6-0.2%.^1,2^ Less frequently, CAF can develop after a variety of cardiovascular treatments as a result of iatrogenic damage. About 90% of cases of CAF are congenital.^3^ Persistence of fetal sinusoids that perfuse the primary myocardium may cause fistulous communicationbetween the coronary arteries and cardiac chambers.

 Similar to other congenital heart diseases, typical CAFs are usually asymptomatic in childhood but adult patients are usually symptomatic. the majority of CAFs are asymptomatic and discovered by chance during imaging or angiography. In symptomatic patients, the most common symptom is secondary heart failure, which is associated with left-to-right shunt overload, ischemia secondary to coronary steal phenomenon, arrhythmia, fistula rupture or thrombosis, and infective endocarditis.^4^

 Common treatment for CAF is surgical closure of the fistula. In 1983, Reidyet al^5^ first introduced transcatheter fistula closure, which was introduced as an alternative to fistula surgery. With shorter hospital stays and recoveries associated with it, transcatheter CAF closure has proven to be a viable option to surgical closure. Several modest single-center studies have also reported its efficacy and safety.^6,7^ However, the reported experience is still somewhat few because to the rarity of CAF. As a result, it is unknown which anatomical and procedural factors could cause CAF difficulties, recanalization, or failed closure.The aim of this study is to investigate the major cardiovascular complications and outcomes of patients who underwent transcatheter closure of CAF.

## Material and Methods

###  Patients

 Our study retrospectively assessed all congenital CAF patients who had transcatheter fistula closure at Rajaie Cardiovascular Medical and Research Center in Tehran from 2015-2019. Patients who underwent catheter closure of symptomatic or large CAF were included in this study. An echocardiogram was performed for all patients before catheterization to evaluate the fistula.Patient demographics, symptoms, physical examination findings, and diagnostic test results were documented for each patient. In this study, coronary angiography and anatomical features of each CAF were evaluated. All information was obtained from patients and their follow-ups were registered to the clinic without publishing the names of the patients. This study was approved by the ethics committee of the Iran University of Medical Sciences (IR.IUMS.).

###  Procedure

 The techniques described in Jama et al study have been applied to the catheter closure of CAF in all patients.^8^ Briefly, access was performed with femoral arterial and/or venous catheters. Bolus heparin (60-100 U/kg) is given to patients after access is secured. Guidewires, microcatheters, or guiding catheters were used to gain access to the fistula after the engagement of the coronary arteries and localization of the CAF origin and terminal site. Ligation was performed using retrograde (arterial), antegrade (venous), or both methods using arteriovenous loop formation. Closed devices were selected based on the size and complexity of the fistula. The devices used in this study included coils, VSD occlude and PDA occlude.Coronary angiography was done right away after the device was deployed to check for any residual flow. Additional devices were applied in patients with large residual flow and amenable anatomy until a satisfactory closure was obtained. All patients received dual antiplatelet therapy for 6 months. Due to concerns about thrombosis from the flow stagnation, long-term anticoagulation was advised in certain patients with significant CAF.

###  Follow up

 The results of follow-up angiograms, noninvasive imaging, and electrocardiograms were examined along with medical records. Acute procedural success, as determined by no or minimal residual flow on post deployment coronary angiography, was the primary outcome. The rate/degree of recanalization for patients who underwent follow-up invasive angiograms following successful index closure were secondary outcomes, along with complications (acute and mid/long-term), survival at 1 and 5 years, and other factors. Grades for residual flow were large, small, and trivial. Small residual flow is defined as residual flow that is less than 50% of the original CAF size, trivial flow is defined as minimal or non-important residual flow, and large residual flow is defined as residual flow that is higher than 50% of the original CAF size. Both procedural and spontaneous myocardial infarctions (MIs) were classified as MIs. Anatomical and procedural traits of individuals failing to close, incurring major complications (such as MIs), or recanalization on angiography were examined in attempt to find potential risk factors for poor outcomes.

###  Statistical analysis

 In the case of nominal variables, data are expressed as a frequency or percentage, while in the case of continuous variables; data are expressed as a mean + standard deviation (SD). We used a paired t test to compare the parameters before and after the procedure. A *P* < 0.05 value was considered statistically significant.

## Result

 In this study, we evaluated 28 patients with CAF during 2015-2020. Characteristic of these patients showed in Table 1. In addition, no one had endocarditis. In the injection immediately after theprocedure, all patients except one patient who failed the procedure had no significant residue. (Of course, it should be noted that the amount of fluid remaining in the injection immediately after the procedure was high due to the use of high amounts of heparin and ACT.) The patient who had an unsuccessful procedure had a fistula with a size of 18 mm and the type of CAF was Distal and the recipient approach was used. Due to the failure of the wire to pass, the patient’s procedure was unsuccessful and a surgical plan was considered.

**Table 1:** characteristics of patients

**Variable**		
Age (year), mean ± SD		53.6 ± 12.8
Gender, n (%)	Male	12 (42.9%)
	Female	16 (57.1%)
Symptoms, n (%)	Chest pain	9 (32.1%)
	Dyspnea	19 (67.9%)
CFT type, n (%)	Proximal	23 (82.1%)
	Distal	5 (17.9%)
Fistula origin, n (%)	RCA	11 (39.3%)
	LAD	9 (32.1%)
	LCX	5 (17.9%)
	Diagonal	2 (7.1%)
	LMCA	1 (3.6%)
Fistula drainage, n (%)	PA	23 (82.1%)
	RA	3 (10.7%)
	SVC	1 (3.6%)
	LA	1 (3.6%)
Approach, n (%)	Antegrade	5 (17.9%)
	Retrograde	22 (78.6%)
	both	1 (3.6%)
Device type, n (%)	Coil	23 (82.1%)
	PDA occluders	2 (7.1%)
	VSD occluders	1 (3.6%)
	PDA occluders + VSD occluders	1 (3.6%)
	None	1 (3.6%)
Number of devices, n (%)	One device	7 (25%)
	Two devices	14 (50%)
	Three devices	4 (14.3%)
	Four devices	1 (3.6%)
	No device	1 (3.6%)
Mean number of devices per CFA, mean		1.82
Fistula diameter (mm), median (min-max)		3.87 (2-20)
Acute procedural success, n (%)		27 (96.4%)
Residual flow grade after procedure	Moderate	4 (14.3%)
	Mild	6 (21.4%)
	No/trivial	17 (60.7%)

LMCA: Left main coronary artery; LAD: left anterior descending artery; LCX: left circumflex artery; RCA: right coronary artery; PA: pulmonary artery; LA: left atrium; RA: right atrium; SVC: superior vena cava

 Moderate residual shunt was reported in 4 patients in the injection immediately after cath. During the follow-up, these 4 patients were all asymptomatic. In the first patients, fistula size was 20mm and the shunt was closed with Occlutech 14x18 mm, CT angiography was performed 6 months after the procedure, and there was no residue. In second patient, the size of the fistula was 14 mm, which was closed with two devices (VSD occlude 12*6, PDA occlude 9*6). This patient underwent re-angiography 8 months later. The amount of residual shunt was reported as mild. The patient’s Pulmonary flow (QP)/ Systemic flow (QS), which was 1.6 before the initial procedure, decreased to 1.2. In third patient, fistula size was 20mm, the patient’s shunt was closed with a Microplex coil (size: 68*24). In the patient’s follow-up echo, the residual shunt was trivial. In fourth patient, the size of the fistula was 10 mm, and the patient’s shunt was closed with an Amplatzer size 8 mm. In the follow-up echo, the patient had no residue and the patient’s EF had increased.

 All patients were asymptomatic during follow-up and remained asymptomatic in echocardiography follow-up of the patients, no significant residue was observed.

**Figure 1:**
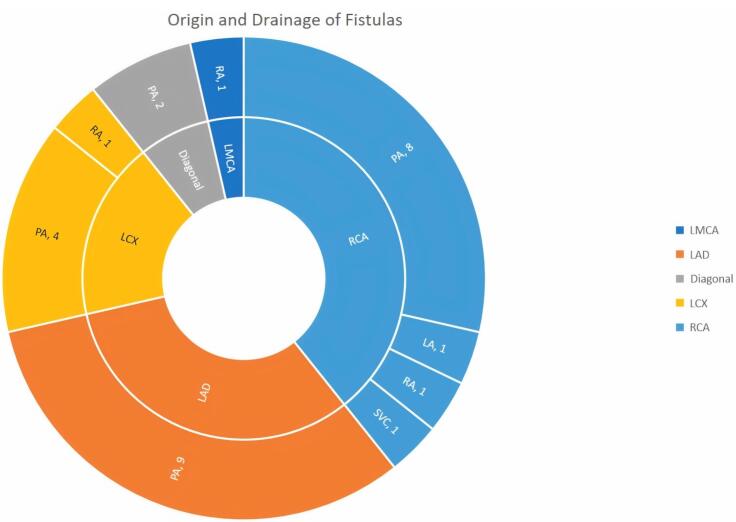
Origin (Inner circle), drainage (Outer circle)and number of CAFs (LMCA: Left main coronary artery; LAD: left anterior descending artery; LCX: left circumflex artery; RCA: right coronary artery; PA: pulmonary artery; LA: left atrium; RA: right atrium; SVC: superior vena cava)

**Figure 2:**
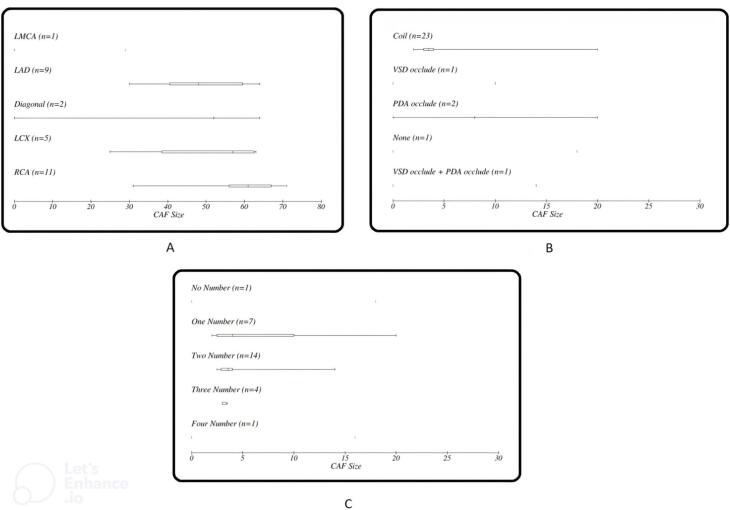
Frequency of CAF size based on (A) initiation, (B) occlude device and (C) the number of occlude device

**Figure 3:**
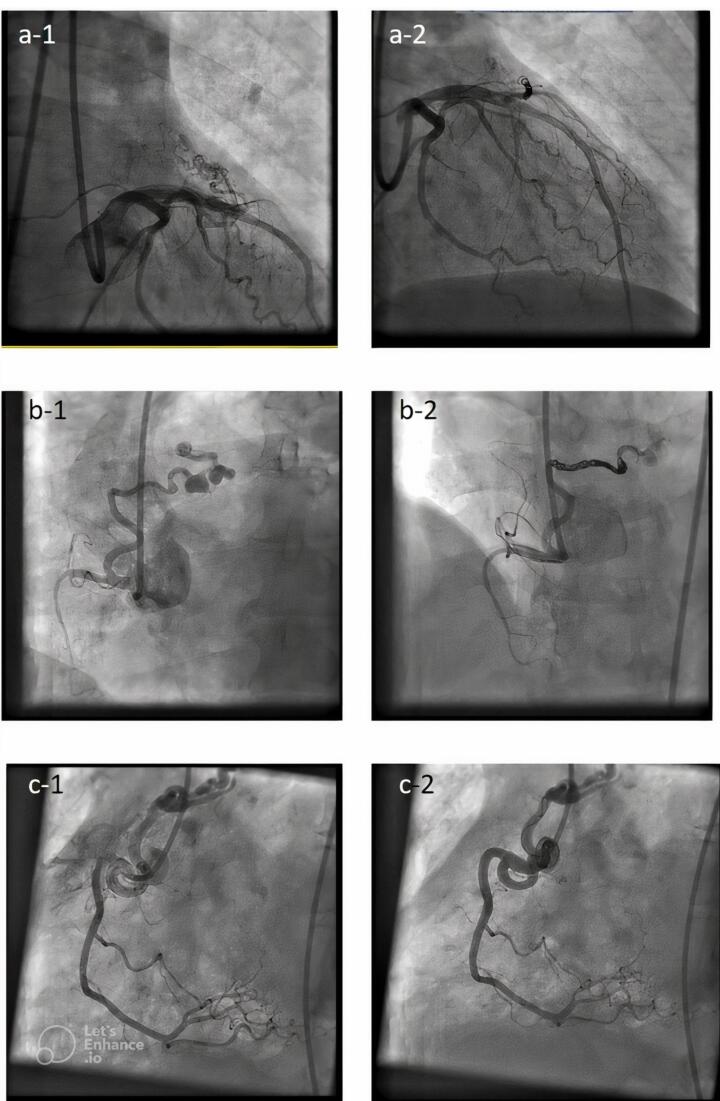
Angiographic images of 3 cases before and after closing congenital coronary fistulas. (a-1) shows coronary artery fistula originated from LAD (Left anterior descending)and drained into PA (Pulmonary artery) before closure and (a-2) shows fistula in same patient after closure. (b-1) shows coronary artery fistula originated from RCA (Right coronary artery)and drained into PA before closure and (b-2) shows fistula in same patient after closure. (c-1) shows coronary artery fistula originated from RCA And drained into PA before closure and (c-2) shows fistula in same patient after closure

 As seen in Figure 1 right coronary artery RCA was the most frequent origin of fistula and most of the fistula drain into the PA. In Figure 2 we showed the frequencies of CAF size based on origin, device type and number of devices that used. In Figure 3, angiographic images of three cases have been showed.

 In follow-up of the patients, 4 of 28 patients underwent follow up CT angiography after 6 months from which only 1 patient had dissection reportedly, as a result of which the flow of the fistula was significantly reduced, and the perfusion scan did not show any evidence of ischemia. Also, none of the studied patients had STEMI (based on ECG after the procedure and clinical follow-up), CVA, infection, peripheral artery thrombosis, device migration, readmission, or death.

## Discussion

 CAFs are uncommon heart defects that can worsen over time and cause serious symptoms. The majority of treatment for large and/or symptomatic CAF is surgical or transcatheter closure, with transcatheter closure frequently being the preferred method in patients with amenable anatomy and no concurrent need for surgery. However, due to the rarity and heterogeneity of CAF, there is little evidence evaluating the effects of anatomical and procedural variables on outcomes. Experience with transcatheter closure is very limited. According to previous studies, the RCA is the most common initiation of CAFs (approximately 50% of cases). Then left anterior descending artery (LAD) artery is 35 to 40% of cases and left circumflex artery (LCX) artery is 5 to 20% of cases respectively.^9^ Nearly 90% of fistulas are drained to low-pressure venous flow, which is the most common location of the right ventricle (41% of cases), followed by the right atrium in 26% of cases, the pulmonary artery in 17% of cases, coronary sinus in 7% of cases, left atrium 26% of cases, left ventricle 3% of cases, and superior vena cava 1% of cases.^10^ The results of our study were in line with these results and RCA is the most common place of origin of CAF.

 In the study of Ouchi et al, the prevalence of CAF was reported to be 0.91%, and unlike previous studies and our study, LAD is the most common origination of congenital CAF (67.8%) and pulmonary artery is the most common drainage site of fistulas (82.3%).^11^ In our study, in terms of the origin of the fistula, the number of fistulas with LAD and diagonal origin was equalto RCA, and pulmonary artery was the most common drainage site of congenital fistulas.

 According to the 2008 ACC/AHA guideline, closure of congenital CAF in all cases of large congenital CAF, even if they are asymptomatic, and in symptomatic cases of small and medium congenital fistulas, should be performed by surgery or transcatheter. Fistula closure is not recommended in cases of asymptomatic small size fistula. It emphasizes an individualized closure approach at experienced centers in the updated 2018 ACC/AHA guidelines.^12^ It is recommended by Mavroudis et al to close congenital CAF by coil only when there are no multiple fistulas, if there is a single and narrow drain site, if there is no large vascular branch, and if there is no danger of rebleeding.^13^ While many congenital CAF have been closed by microcoils in recent years, many devices have been used to close fistulas.

 Retrograde approach is often used in percutaneous closure of congenital CAF. In our study, both approaches were used to close the fistula. The retrograde approach has advantages over the antegrade approach, and in cases where the fistula drainage is near the coronary sinus, it is better to use the retrograde approach. In the retrograde method, there is no need to create an arteriovenous loop, but there is a possibility of damage to peripheral vessels. Therefore, the need for thrombolytic treatment should be considered. The most important advantage of the retrograde approach is familiarity with it, but in some cases, such as fistulas with small feeding vessels, it is better to use the antegrade approach.^7^ Using a larger catheter and a straighter course of the catheter and avoiding access from the femoral artery are the advantages of the antegrade method. However, there is a risk of device embolization in the antegrade approach due to lack of flow control.^14^ Choosing the type of device and its transfer technique is also effective in the success of the procedure and its complications. In our study, different devices with different sizes were used, and in the end, the procedure was unsuccessful in 1 patient due to the failure of the wire to pass through the fistula.

 Previous studies have mentioned multiple complications associated with Transcatheter closure of CAF, including unrecovered device embolism, transient ischemia, fistula dissection, cardiomyopathy, and transient arrhythmia.^15^ In our study, only one case of fistula dissection was reported, which indicates the safety of this procedure.The algorithm of long-term follow-up of congenital coronary fistula after inversion is not very clear. Due to the possibility of recanalization, residual flow, stable coronary artery dilatation, late thrombosis and myocardial ischemia, long-term follow-up should be done.^16^ Therefore, although many patients are asymptomatic after the intervention, they should be followed up due to possible long-term complications. In follow-up, clinical examination with electrocardiogram and echocardiography should be done, and when the evidence is in favor of myocardial ischemia, the condition of myocardial perfusion should be checked.^17^ It is better to follow up patients 1, 3, 6, and 12 months after the procedure and then annually.In our study, patients were followed up in several time intervals.It is better to perform coronary angiography in symptomatic patients who suspect complications.^18^ Since the necessity of performing coronary angiography in long-term follow-up in asymptomatic patients is not clear, therefore asymptomatic patients should undergo follow-up with non-invasive modalities. CT angiography and cardiac MRI can provide valuable data about the remaining flow.^19^ However, it should be noted that CT angiography is not a suitable method for examining fistulas originating from distal coronary vessels with a small vascular structure and course between the coronary artery and the heart chamber.^20^

## Conclusion

 Although closing congenital coronary fistulas by surgical ligation method was a standard method in the past, the use of special techniques and new devices has made it possible to use the transcatheter approach in many patients with suitable anatomy as the first line of treatment. It has been found that CAF closure through the transcatheter approach can be a successful procedure with low morbidity for certain patients.The CAF should be closely followed after closure to ensure there is no recanalization and to detect potential complications, as a repeat closure may be required due to the risk of recanalization.

## Competing Interests

 All authors declare no competing interests.

## Ethical Approval

 Additional informed consent was obtained from all patients.

 Ethics code approval from Iran University of Medical Sciences Ethics committee: 15391
